# Responsiveness of PROMIS and Patient Health Questionnaire (PHQ) Depression Scales in three clinical trials

**DOI:** 10.1186/s12955-021-01674-3

**Published:** 2021-02-04

**Authors:** Kurt Kroenke, Timothy E. Stump, Chen X. Chen, Jacob Kean, Teresa M. Damush, Matthew J. Bair, Erin E. Krebs, Patrick O. Monahan

**Affiliations:** 1 Department of Medicine, Indiana University School of Medicine, Indianapolis, IN USA; 2 Regenstrief Institute, Inc, 1101 West 10th St., Indianapolis, IN 46202 USA; 3 Department of Biostatistics, Indiana University Fairbanks School of Public Health and School of Medicine, Indianapolis, IN USA; 4 Indiana University School of Nursing, Indianapolis, IN USA; 5 Department of Population Health Sciences, University of Utah School of Medicine, Salt Lake City, Utah, USA; 6 VA Health Services Research and Development Center for Health Information and Communication, Indianapolis, IN USA; 7 Center for Chronic Disease Outcomes Research, Minneapolis VA Health Care System, Minneapolis, MN USA; 8 University of Minnesota Medical School, Minneapolis, MN USA

**Keywords:** PROMIS, PHQ-9, Depression, Responsiveness, Sensitivity to change, Psychometrics

## Abstract

**Background:**

The PROMIS depression scales are reliable and valid measures that have extensive normative data in general population samples. However, less is known about how responsive they are to detect change in clinical settings and how their responsiveness compares to legacy measures. The purpose of this study was to assess and compare the responsiveness of the PROMIS and Patient Health Questionnaire (PHQ) depression scales in three separate samples.

**Methods:**

We used data from three clinical trials (two in patients with chronic pain and one in stroke survivors) totaling 651 participants. At both baseline and follow-up, participants completed four PROMIS depression fixed-length scales as well as legacy measures: Patient Health Questionnaire 9-item and 2-item scales (PHQ-9 and PHQ-2) and the SF-36 Mental Health scale. We measured global ratings of depression change, both prospectively and retrospectively, as anchors to classify patients as improved, unchanged, or worsened. Responsiveness was assessed with standardized response means, statistical tests comparing change groups, and area-under-curve analysis.

**Results:**

The PROMIS depression and legacy scales had generally comparable responsiveness. Moreover, the four PROMIS depression scales of varying lengths were similarly responsive. In general, measures performed better in detecting depression improvement than depression worsening. For all measures, responsiveness varied based on the study sample and on whether depression improved or worsened.

**Conclusions:**

Both PROMIS and PHQ depression scales are brief public domain measures that are responsive (i.e., sensitive to change) and thus appropriate as outcome measures in research as well as for monitoring treatment in clinical practice.

*Trial registration* ClinicalTrials.gov ID: NCT01236521, NCT01583985, NCT01507688

## Background

Depression is the most common mental health disorder in both clinical practice and the general population, a major contributor to disability and health care costs, and an important cause of morbidity as well as early mortality [1]. Because the assessment and monitoring of depression relies principally on patient-reported symptoms, reliable and valid scales are essential for both research and clinical practice. The National Institutes of Health has made substantial investments in developing and testing the Patient-Reported Outcomes Measurement Information System (PROMIS) measures to assess symptoms and functional domains that cut across a number of medical and psychological conditions [2].

Initially developed and validated in the general population, PROMIS measures are increasingly being tested in clinical settings. However, there are substantial gaps in understanding the performance of PROMIS measures in patients. One particularly important psychometric characteristic is a scale’s responsiveness (alternatively called sensitivity to change) which focuses on a measure’s ability to detect changes over time [3]. A responsive measure is essential for clinical trials and other longitudinal studies to minimize the risk of false negative conclusions as well as to potentially reduce sample size and study costs. Responsiveness is also critical in clinical practice where the purpose is to detect clinically meaningful change over time in order to monitor and, if necessary, adjust treatment.

PROMIS measures draw upon item banks that are calibrated using item response theory and include large numbers of questions that collectively represent a well-defined, unidimensional construct. Individual questions from these large banks can then be extracted, using various strategies, to create unique short forms of that measure [2]. These short forms can be static (i.e., the same items used in a fixed-length scale), or they can be constructed adaptively in real time based on the respondent’s answers to previous questions, known as computer adaptive testing (CAT). Although CAT may require a few less items than fixed-length forms to obtain comparable precision, the small increase in efficiency may not be sufficient to justify the added technical requirements for CAT administration.

Four PROMIS fixed-length depression scales are the focus of this study, which includes one with 4 items, one with 6 items, and two with 8 items. Fixed-length scales were chosen rather than CAT administration because in many clinical and research settings fixed-length scales are more feasible to administer and produce approximately comparable results to CAT. For this reason, fixed-length scales have been offered as a viable option by PROMIS developers [4].

Only a few studies have examined PROMIS depression scale responsiveness. These studies have several limitations, including studying only a single sample [5–8], no comparison to a legacy or other anchor measure[7], and focusing only or principally on CAT rather than fixed-length PROMIS measures [5, 7, 9]. Given the limitations of previous studies, our study purpose was to evaluate responsiveness of the four fixed-length PROMIS depression scales, and compare their responsiveness to legacy depression measures using three clinical samples. It should be noted that scores on these self-report scales represent depressive symptom severity rather than a depressive disorder diagnosis; the latter requires a clinical assessment.

## Methods

### Design and participants

Data were analyzed from three randomized controlled trials (RCTs) conducted between 2012 and 2017. Trial details were provided in a previous report of the minimally important differences and severity thresholds for the PROMIS depression measures [10]. Briefly, the study sample includes 651 patients who had complete psychometric data on depression measures (Table 1). Sample 1 (CAMEO trial) consisted of 153 primary care patients participating in an RCT to compare the effectiveness of pharmacological versus cognitive-behavioral treatment for chronic low back pain. Sample 2 (SPACE trial) consisted of 240 primary care patients participating in a pragmatic RCT comparing opioid therapy versus non-opioid medication therapy for chronic back pain or hip or knee osteoarthritis pain. Sample 3 (SSM trial) consisted of 258 stroke survivors participating in an RCT evaluating the efficacy of a stroke-self-management program. Samples 1 and 2 were enrolled from Veterans Administration (VA) primary care clinics, and Sample 3 comprised both Veteran and non-Veteran patients. Data were collected from baseline and follow-up interviews administered by trained research personnel. Follow-up assessments were conducted 6 months after baseline for Sample 1 and 3 months after baseline for Samples 2 and 3. The studies were approved by the Indiana University Institutional Review Board.

**Table 1:** Characteristics of samples in three randomized controlled trials (RCTs)

Clinical population	Sample 1 CAMEO RCT (N_1_ = 153)	Sample 2 SPACE RCT (N_2_ = 240)	Sample 3 SSM RCT (N_3_ = 258)
	Chronic low back pain	Chronic musculoskeletal pain	Stroke survivors
Recruitment setting	Primary care	Primary care	Neurology
Age, mean (SD)	58.1 (9.3)	58.3 (13.7)	61.7 (10.8)
Male, n (%)	140 (91.5)	208 (86.7)	209 (81.0)
Race, n (%)			
White	111 (72.5)	207 (86.2)	166 (64.3)
Black	37 (24.2)	18 (7.5)	78 (30.2)
Other	5 (3.3)	15 (6.3)	14 (5.4)
Education, n (%)			
Less than high school	8 (5.2)	6 (2.5)	31 (12.2)
High school	49 (32.0)	71 (29.6)	85 (33.3)
Technical school or some college	74 (48.4)	103 (42.9)	80 (31.4)
College degree or greater	22 (14.4)	60 (25.0)	59 (23.1)
Marital status, n (%)			
Married	81 (52.9)	135 (56.5)	135 (52.5)
Divorced	43 (28.1)	60 (25.1)	68 (26.5)
Other	29 (19.0)	44 (18.4)	54 (21.0)
PROMIS T-scores, mean (SD)			
Depression 4-item	53.5 (9.9)	50.3 (9.1)	51.3 (9.2)
Depression 6-item	53.2 (10.3)	49.9 (9.5)	50.5 (10.0)
Depression 8-item	53.0 (10.2)	49.6 (9.5)	50.3 (9.9)
Depression short-form	53.0 (10.3)	49.7 (9.7)	50.0 (10.3)
PHQ-9 depression score, mean (SD)	11.1 (6.2)	6.2 (5.0)	7.7 (6.2)
Cross-sectional Global Ratings of Depression (0–4), mean (SD)	2.5 (1.0)	2.0 (0.9)	1.9 (1.0)
DSM-V depressive disorder, n (%)			
Major depression	68 (44.4)	36 (15.0)	66 (25.6)
Minor depression	21 (13.7)	23 (9.6)	21 (8.1)
Disability days in the past 4 weeks, mean (SD)	16.3 (8.6)	10.3 (9.0)	5.1 (7.7)

*CAMEO* CAre Management for the Effective use of Opioids trial, *SPACE* Strategies for Prescribing Analgesics Comparative Effectiveness trial, *SSM* Stroke survivor Self-Management trial

## Measures

### PROMIS Depression Scales

We evaluated four fixed-length PROMIS depression scales: the original 8-item depression Short Form (8b), and the 4-item (4a), 6-item (6a) and 8-item (8a) depression scales from the PROMIS profiles (a collection of short forms containing a fixed number of items from key PROMIS domains). Items are nested in the latter three scales: the 6a scale adds two items to the 4a scale, and the 8a scale adds two items to the 6a scale. The 8a and 8b scales share 7 items in common and 1 unique item each. For each scale, respondents are asked how often in the past 7 days they have experienced specific depression symptoms, using a 5-point ordinal rating scale of “Never,” “Rarely,” “Sometimes,” “Often,” and “Always.” Raw score totals are converted to an item response theory-based T-scores. A T-score of 50 is the average for the United States general population with a standard deviation (SD) of 10. A higher T-score represents greater depression severity. Cronbach’s alphas for baseline PROMIS raw scores in the three trials ranged from 0.89 to 0.95.

### Patient Health Questionnaire 9-item (PHQ-9) and 2-item (PHQ-2) Depression Scales

The PHQ-9 is among the best-validated and widely-used depression scales in both clinical practice and research [11, 12]. The PHQ-9 [13] includes 1 item for each of the 9 DSM-V criterion symptoms used in diagnosing major depression. Respondents are asked how much in the past 2 weeks they have been bothered by each symptom, with the response options being “Not at all”, “Several days”, “More than half the days”, and “Nearly every day.” Scores range from 0 to 27 with higher scores indicating greater depression severity. The Cronbach’s alpha for baseline PHQ-9 scores in the three trials ranged from 0.76 to 0.85. The PHQ-2 comprises the first two items of the PHQ-9 that capture depressed mood and anhedonia. It is scored 0 to 6 and has been validated as an ultra-brief screening tool [12] with some evidence of responsiveness [13, 14].

### SF-36 Mental Health Scale

The SF-36 Mental Health scale was administered only in Sample 1 (CAMEO trial). The scale consists of five items with each item scored from 1 (not at all) to 5 (extremely) scale over the past four weeks. Responses from the five items are summed and then transformed to a 0–100 scale where a lower number represents more severe symptoms. The scale has demonstrated good operating characteristics as a depression screener as well as sensitive to change in longitudinal studies [15, 16].

### Prospective global rating of change

The prospective global rating of change is the difference between an individual’s cross-sectional global rating of mood at two time points (baseline minus follow-up) [17]. Because the cross-sectional global rating is on a 5-point scale ranging from 0 (“Not unhappy or down at all”) to 4 (“Very severely unhappy or down”), change scores have a possible range of − 4 to + 4, where negative numbers indicated worsening mood and positive numbers improved mood. For example, a patient who reported being “severely unhappy or down” at baseline and “mildly unhappy or down” at follow-up would have a + 2 change (3 − 1), whereas a patient who reported being “moderately unhappy or down” at baseline and “severely unhappy or down” at follow-up would have a − 1 change (2 − 3). Change scores were collapsed into three categories of better (+ 1 to + 4), same (0), and worse (− 1 to − 4). We used this prospective anchor to overcome potential recall and reconstruction bias related to the retrospective global rating of change [18]. A few studies have suggested, compared to the retrospective global rating of change, that the prospective global rating of change may be less influenced by post-treatment status [18, 19].

### Retrospective global rating of change

The retrospective global rating of change assesses overall clinical response from the participant’s perspective [20]. At follow-up, participants were asked to rate their mood change compared to their mood at baseline assessment. Change in mood is rated on a 7-point scale with the following response options: − 3 (much worse), − 2 (moderately worse), − 1 (a little worse), 0 (no change), + 1 (a little better), + 2 (moderately better), or + 3 (much better). Based on the rating, participants were further categorized into three groups, improved (+ 1 to + 3), unchanged (0), and worsened (− 1 to − 3). The retrospective global rating of change has been widely used to assess responsiveness of patient-reported outcome measures [3, 16].

### Statistical analysis

We evaluated comparative responsiveness for all four PROMIS scales and legacy measures (i.e., PHQ-9, PHQ-2, and SF-36 Mental Health). Data from each of the three trials were analyzed separately rather than pooled, because the three trials involved different clinical populations, study interventions, and follow-up timeframes. We used both prospective and retrospective global ratings of change for mood as the anchors (i.e., criteria) to identify patients who had changed since baseline. Specifically, patients were categorized into three groups based on global ratings of mood change: better, same, and worse. Both within-group and between-group responsiveness to change were evaluated.

### Within-group responsiveness

For within-group responsiveness, we estimated the amount of change over time within each global rating of depression change group (i.e., better, same, and worse). The standardized response mean (SRM) was used as the effect size measure of within-group responsiveness to change. The SRM is the ratio of the mean change to the standardized deviation (SD) of change, and is calculated using the formula (mean baseline score − mean follow-up score)/(SD of change score). We also calculated 95% confidence intervals for the SRMs with a bootstrapping procedure. SRM values of 0.2, 0.5, and 0.8 represent thresholds for small, moderate and large changes, respectively [3, 21]. Some researchers suggest an absolute SRM value ≥ 0.3 indicates responsiveness [22].

### Between-group responsiveness

For between-group responsiveness, we compared the amount of change between global rating of change groups. First, we used omnibus ANOVA tests to compare mean change scores across global rating of change groups (i.e., improved, unchanged, and worsened). For this analysis, both retrospective and prospective rating of change groups were used as anchors. We used post-hoc Tukey–Kramer pairwise tests to compare the three groups and controlled for family-wise Type 1 error at 0.05.

Second, we used receiver-operating characteristic curve analyses to further quantify a measure’s ability to detect improvement. Area under the curve (AUC) is the probability of correctly discriminating between patients who have improved and those who have not. The AUC values range from 0.5 (the same as chance) to 1.0 (perfect discrimination). We calculated the AUC for each depression measure using retrospective and prospective global ratings of change as the anchors. For the retrospective anchor, we evaluated each measure’s ability to detect *any* improvement (“a little better”, “moderately better”, or “very much better”) as well as *moderate* improvement (“moderately better” or “very much better”). For the prospective anchor, we evaluated each measure’s ability to detect *any* improvement (+ 1 to + 4) as well as *moderate* improvement (+ 2 to + 4). To determine if depression scales differed in their ability to detect improvement, we also statistically compared AUC values between measures [20, 23].

## Results

### Demographic and clinical characteristics

For all three samples, participants were mostly male, non-Hispanic, white, married, and had some college education (Table 1). Mean PHQ-9 scores indicated that sample 1 had moderate and samples 2 and 3 had mild levels of depressive symptoms. The proportion of patients who met DSM-V criteria for major or minor depression in the 3 studies was 58.1%, 24.6%, and 33.7%, respectively.

### Within-group responsiveness

**Fig. 1:**
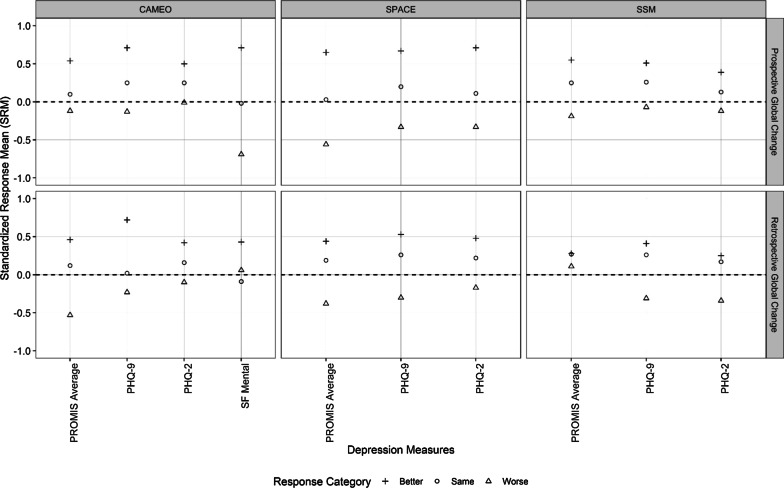
Comparative standardized response means (SRMs) between depression measures across trials

In Fig. 1, within-group effect size estimates (i.e., SRMs) were plotted for the PROMIS depression and legacy measures across the three trials. This figure provides an overview of comparative within-group responsiveness across the depression measures. Tables 2 and 3 complement Fig. 1 by presenting the unstandardized change scores and SRMs with confidence intervals for the prospective and retrospective anchors, respectively.

**Table 2:** Responsiveness of depression measures by prospective global rating of change for mood

Depression change	Mean SRM	CAMEO				SPACE				SSM			
		Score change*	SRM^†^	(95% CI)	P^‡^	Score change*	SRM^†^	(95% CI)	P^‡^	Score change*	SRM^†^	(95% CI)	P^‡^
PROMIS 4-item					**.008**				** < .0001**				** < .0001**
Better	.54	3.58	.48	(.23, .75)	.019	5.12	.59	(.38, .81)	< .0001	4.81	.56	(.32, .82)	.005
Same	.10	− 0.17	− .02	(− .27, .25)	–	− 0.14	− .02	(− .21, .17)	–	1.41	.27	(.10, .44)	–
Worse	− .27	− 1.09	− .12	(− .55, .32)	.87	− 3.38	− .55	(− .95, − .19)	.122	− 1.55	− .16		.072
PROMIS 6-item					**.008**				** < .0001**				** < .0001**
Better	.58	4.13	.54	(.29, .80)	.039	5.85	.64	(.42, .87)	< .0001	5.17	.55	(.33, .79)	.003
Same	.13	0.72	.13	(− .14, .41)	–	0.16	.03	(− .16, .21)	–	1.35	.23	(.06, .41)	–
Worse	− .28	− 1.03	− .11	(− .54, .33)	.61	− 2.95	− .52	(− .94, − .12)	.186	− 1.94	− .21	(− .53, .12)	.055
PROMIS 8-item					**.007**				**< .0001**				**< .0001**
Better	.59	4.22	.56	(.31, .83)	.037	5.93	.65	(.43, .88)	< .0001	5.16	.55	(.33, .79)	.002
Same	.14	0.76	.13	(− .13, .39)	–	0.19	.03	(− .15, .21)	–	1.39	.26	(.09, .44)	–
Worse	− .27	− 1.00	− .11	(− .54, .34)	.61	− 3.12	− .53	(− .95, − .14)	.141	− 1.56	− .17	(− .48, .16)	.082
PROMIS SF					**.009**				**< .0001**				**< .0001**
Better	.60	4.22	.56	(.31, .84)	.077	6.51	.70	(.47, .95)	< .0001	5.26	.54	(.31, .79)	.003
Same	.16	1.10	.17	(− .09, .44)	–	0.45	.08	(− .11, .26)	–	1.33	.22	(.05, .39)	–
Worse	− .33	− 1.35	− .14	(− .58, .30)	.40	− 3.57	− .65	(− 1.2, − .19)	.060	− 1.94	− .20	(− .51, .12)	.068
PROMIS average													
Better	.58		.54				.65				.55		
Same	.13		.10				.03				.25		
Worse	− .29		− .12				− .56				− .19		
PHQ-9					**.002**				**< .0001**				**.001**
Better	.63	3.56	.71	(.43, 1.0)	.026	2.52	.67	(.48, .87)	.002	3.23	.51	(.27, .76)	.014
Same	.24	1.07	.25	(− .02, .56)	–	0.69	.20	(.02, .40)	–	1.13	.26	(.09, .43)	–
Worse	− .18	− 0.73	− .13	(− .60, .31)	.34	− 1.45	− .33	(− .84, .10)	.039	− 0.30	− .07	(− .42, .26)	.28
PHQ-2					**.163**				** < .0001**				**.007**
Better	.53	0.78	.50	(.25, .75)	.44	0.93	.71	(.53, .89)	< .0001	0.81	.39	(.14, .66)	.045
Same	.16	0.39	.25	(.− .01, .51)	–	0.12	.11	(− .08, .33)	–	0.19	.13	(− .04, .30)	–
Worse	− .24	0.00	− .01	(− .49, .41)	.63	− 0.48	− .33	(− .97, .14)	.143	− 0.24	− .12	(− .42, .22)	.38
SF-36 Mental					** < .0001**								
Better		15.18	.71	(.50, .94)	< .0001								
Same		− 0.22	− .02	(− .28, .25)	–								
Worse		− 9.55	− .69	(− 1.5, − .17)	.076								

Total N (better, same, worse) with baseline and follow-up data in CAMEO = 135 (55, 58, 22); in SPACE = 223 (87, 114, 22); and in SSM = 239 (70, 131, 38)

*Score change = baseline—follow-up (positive score indicates improvement, and negative score indicates worsening)

^†^SRM = (baseline − follow-up)/SD change score;

^‡^Bolded p-values are from omnibus ANOVA tests comparing change scores among the three groups. Other p values were derived from pairwise comparisons of change scores between better vs. same, same vs. worse, and better vs. worse, and were adjusted for multiple comparisons using the Tukey–Kramer procedure. Since all better vs. worse pairwise comparisons were significant when the omnibus test was significant, only better vs. same and same vs. worse p-values are reported in this table

**Table 3:** Responsiveness of Depression Measures by Retrospective Global Rating of Change for Mood

Depression change	Mean SRM	CAMEO				SPACE				SSM			
		Score change*	SRM^†^	(95% CI)	P^‡^	Score change*	SRM^†^	(95% CI)	P^‡^	Score change*	SRM^†^	(95% CI)	P^‡^
PROMIS 4-item					**.003**				**.002**				**.87**
Better	.35	2.71	.37	(.16, .58)	.217	3.27	.41	(.22, .60)	.086	2.09	.27	(.10, .45)	1.00
Same	.17	0.37	.07	(− .23, .44)	–	1.04	.15	(− .03, .33)	–	2.08	.29	(.08, .52)	–
Worse	‒.35	− 3.94	− .58	(− 1.2, − .08)	.110	− 3.78	− .64	(− 1.1, − .28)	.050	1.14	.18	(− .28, .69)	.87
PROMIS 6-item					**.0007**				**.018**				**.71**
Better	.39	3.57	.47	(.26, .70)	.102	3.68	.43	(.24, .61)	.113	2.28	.27	(.11, .45)	.94
Same	.19	0.70	.13	(− .18, .51)	–	1.41	.18	(.00, .37)	–	1.89	.25	(.03, .47)	–
Worse	‒.24	− 3.81	− .52	(− 1.2, − .01)	.089	− 2.03	− .30	(− .82, .22)	.269	0.72	.11	(− .35, .61)	.83
PROMIS 8-item					**.0007**				**.016**				**.55**
Better	.41	3.59	.49	(.27, .70)	.135	3.71	.43	(.25, .62)	.112	2.33	.30	(.13, .46)	.99
Same	.21	0.90	.16	(− .15, .53)	–	1.45	.19	(.01, .38)	–	2.15	.29	(.08, .50)	–
Worse	‒.25	− 3.91	− .51	(− 1.1, .02)	.066	− 2.10	− .30	(− .80, .23)	.239	0.30	.05	(− .41, .55)	.60
PROMIS SF					**.0005**				**.014**				**.63**
Better	.42	3.82	.51	(.29, .74)	.085	4.10	.48	(.30, .65)	.096	2.40	.28	(.11, .45)	.85
Same	.19	0.75	.13	(− .18, .48)	–	1.71	.22	(.04, .41)	–	1.78	.23	(.02, .45)	–
Worse	‒.23	− 3.93	− .51	(− 1.2, .03)	.085	− 1.82	− .27	(− .84, .27)	.258	0.63	.10	(− .36, .60)	.84
PROMIS average													
Better	.39		.46				.44				.28		
Same	.19		.12				.19				.27		
Worse	‒.27		− .53				− .38				.11		
PHQ-9					**.0001**				**.007**				**.009**
Better	.55	3.35	.72	(.50, .97)	.002	1.98	.53	(.32, .74)	.127	2.16	.41	(.26, .57)	.385
Same	.19	0.07	.02	(− .30, .34)	–	0.93	.26	(.07, .45)	–	1.22	.26	(.05, .48)	–
Worse	‒.28	− 1.28	− .23	(− .65, .25)	.601	− 1.20	− .30	(− 1.3, .26)	.111	− 1.50	− .31	(− .74, .15)	.082
PHQ-2					**.077**				**.042**				**.088**
Better	.38	0.76	.42	(.21, .65)	.250	0.61	.48	(.29, .66)	.203	0.44	.25	(.08, .42)	.804
Same	.18	0.24	.16	(− .15, .50)	–	0.28	.22	(.03, .42)	–	0.28	.17	(− .06, .41)	–
Worse	‒.20	− 0.11	− .10	(− .59, .39)	.735	− 0.29	− .17	(− .81, .41)	.310	− 0.50	− .34	(− .97, .12)	.183
SF-36 Mental					**.042**								
Better		7.92	.43	(.23, .63)	.038								
Same		− 1.10	− .09	(− .45, .22)	–								
Worse		1.94	.06	(− .50, .48)	.836								

Total N (better, same, worse) with baseline and follow-up data in CAMEO = 138 (79, 41, 18); in SPACE = 224 (89, 120, 15); and in SSM = 238 (136, 82, 20)

* Score change = baseline—follow-up (Positive score: improvement, negative score: worsening)

^†^SRM = (baseline − follow-up)/SD change score;

^‡^Bolded p-values are from omnibus ANOVA tests comparing change scores among the three groups. Other p values were derived from pairwise comparisons of change scores between better vs. same, same vs. worse, and better vs. worse, and were adjusted for multiple comparisons using the Tukey–Kramer procedure. Since all better vs. worse pairwise comparisons were significant when the omnibus test was significant, only better vs. same and same vs. worse p-values are reported in this table

Across the PROMIS depression, PHQ-9 and PHQ-2 scales, the SRM point estimates were generally similar (Figs. 1). In most cases, the confidence interval for one measure included the point estimates of the other measures (Tables 1 and 2), which indirectly suggests statistically comparable within-group responsiveness across these three measures. SRMs for the SF-36 mental health scale differed somewhat from the other measures, although data for this scale was only available from one trial.

Minor differences in SRMs, however, were observed. For example, retrospective anchor analyses in the CAMEO trial (sample 1) found larger absolute SRMs for improvement with the PHQ-9 compared to PROMIS but larger SRMs for worsening with the PROMIS. In contrast, the SSM trial (sample 3) revealed larger SRMs for worsening with the PHQ-9 and PHQ-2.

**Fig. 2:**
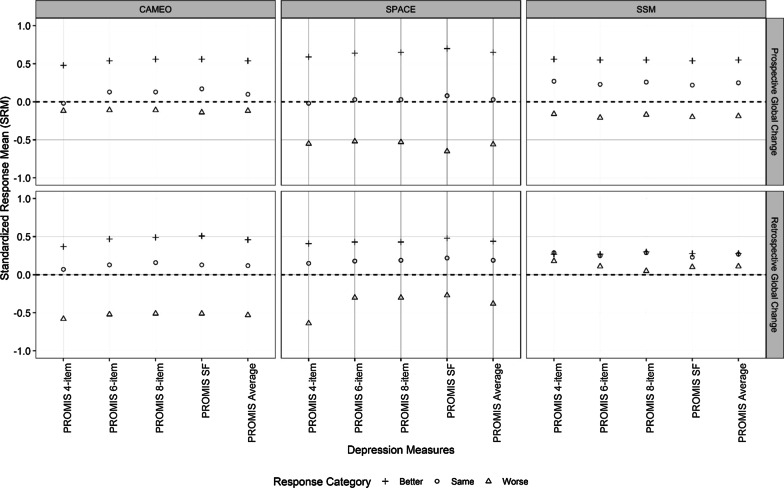
Comparative standardized response means (SRMs) between PROMIS depression short forms of varying lengths

Across the four PROMIS depression scales of varying lengths, the SRMs were relatively comparable (Fig. 2). The mean (median) within-group difference in SRMs between any two PROMIS scales was 0.084 (0.080) using the prospective anchor and within 0.114 (0.070) using the retrospective anchor. Because the SRM estimates for the four PROMIS scales were similar, we reported averages of SRMs across the four PROMIS depression short forms in Fig. 1.

### Between-group responsiveness

As shown in Table 2, all measures successfully detected differences among depression improved, unchanged, and worsened groups when classified by the prospective global rating of change for mood. Omnibus F-tests were all significant (many at *p* < 0.0001) for overall differentiation among change with only one exception (the PHQ-2 in the CAMEO trial). In pair-wise comparisons, scales distinguished better from unchanged in all but two instances (the PROMIS short-form 8b and PHQ-2 in the CAMEO trial). In contrast, scales did not distinguish worse from unchanged except in one instance (the PHQ-9 in the SPACE trial). The mean SRM for the PROMIS average, PHQ-9, and PHQ-2 scores across the CAMEO, SPACE and SSM trials was 0.58, 0.63, and 0.53 for the improved group; 0.13, 0.27, and 0.16 for the unchanged group; and − 0.29, − 0.18, and − 0.15 for the worse group.

When using the retrospective global rating of change anchor (Table 3), the ability of measures to detect differences among the three groups was not quite as strong. Omnibus F-tests were still significant in two of the trials (except the PHQ-2 in CAMEO) but not as highly significant as for the prospective anchor. Moreover, none of the omnibus F-tests were significant in the SSM trial, except for the PHQ-9. The mean SRM using the retrospective anchor for the PROMIS average, PHQ-9, and PHQ-2 scores across the CAMEO, SPACE and SSM trials was 0.39, 0.55, and 0.38 for the improved group; 0.19, 0.18, and 0.18 for the unchanged group; and − 0.27, − 0.28, and − 0.20 for the worse group.

Table 4 shows the results from the AUC analysis for *moderate improvement*. Averaged across the 3 trials, AUCs using the retrospective global change anchor were 0.603 to 0.625 for the PROMIS scales, 0.636 for the PHQ-9, and 0.588 for the PHQ-2. AUCs using the prospective global change anchor averaged 0.745–0.757 for the PROMIS scales, 0.682 for the PHQ-9, and 0.631 for the PHQ-2. Table 5 shows that AUCs for detecting *any improvement* were somewhat lower**.**

**Table 4:** Area under the receiver operating characteristic curve (AUC) for depression measures detecting moderate improvement

Depression measure*	Average accuracy across trials		Accuracy for detecting moderate improvement^†^ Retrospective Global Rating of Change (GRC)						Accuracy for detecting moderate improvement^†^ Prospective Global Rating of Change (GRC)					
	Retro-spective GRC	Pro-spective GRC	CAMEO		SPACE		SSM		CAMEO		SPACE		SSM	
			AUC	(95% CI)	AUC	(95% CI)	AUC	(95% CI)	AUC	(95% CI)	AUC	(95% CI)	AUC	(95% CI)
PROMIS 4-item	.603	.751	.625	(.522–.728)	.640	(.553–.727)	.545	(.473–.617)	.773	(.650–.895)	.819	(.700–.934)	.663	(.488–.838)
PROMIS 6-item	.619	.745	.653	(.553–.753)	.645	(.556–.734)	.560	(.487–.634)	.767	(.642–.892)	.811	(.697–.926)	.657	(.491–.823)
PROMIS 8-item	.610	.751	.632	(.530–.734)	.642	(.555–.728)	.557	(.483–.631)	.751	(.619–.883)	.816	(.702–.929)	.687	(.539–.844)
PROMIS Short-form	.625	.757	.680	(.583–.777)	.638	(.551–.725)	.558	(.484–.631)	.760	(.638–.881)	.836	(.729–.942)	.676	(.514–.838)
PHQ-9	.636	.682	.625	(.526–.724)	.669	(.592–.747)	.614	(.542–.686)	.705	(.568–.841)	.660	(.532–.787)	.681	(.515–.846)
PHQ-2	.588	.631	.587	(.482–.692)	.616	(.537–.695)	.562	(.492–.632)	.609	(.455–.764)	.679	(.553–.806)	.605	(.424–.785)
SF-36 Mental Health	–	–	.580	(.473–.687)	–	–	–	–	.810	(.675–.944)	–	–	–	–

*AUC is probability of correctly discriminating between patients who have improved and those who have not. Any improvement ≥ “a little better”; moderate improvement ≥ “moderately better”

^†^6 month follow-up for CAMEO; 3 months for SPACE and SSM. The proportion of patients reporting moderate improvement by *retrospective GRC* was 25%, 24%, and 42% in CAMEO, SPACE, and SSM, respectively. The proportion reporting moderate improvement by *prospective GRC* was 13%, 11%, and 7% in CAMEO, SPACE, and SSM, respectively

There were no significant differences at P < .01 (using Bonferroni’s correction for multiple comparisons) between any of the retrospective AUC’s. The prospective AUCs were significantly lower for the PHQ-9 (P = .008) and PHQ-2 (P = .004) compared to the PROMIS Short-form (with P = .01 to .02 range compared to the other PROMIS scales) in the SPACE trial and for the PHQ-2 (P = .007) compared to the SF-36 in the CAMEO trial

**Table 5:** Area under the receiver operating characteristic curve (AUC) for depression measures for detecting any improvement

Depression measure*	Average accuracy across trials		Accuracy for detecting any improvement^†^ Retrospective Global Rating of Change (GRC)						Accuracy for detecting any improvement^†^ Prospective Global Rating of Change (GRC)					
	Retro-spective GRC	Pro-spective GRC	CAMEO		SPACE		SSM		CAMEO		SPACE		SSM	
			AUC	(95% CI)	AUC	(95% CI)	AUC	(95% CI)	AUC	(95% CI)	AUC	(95% CI)	AUC	(95% CI)
PROMIS 4-item	.565	.658	.603	(.510–.697)	.586	(.512–.660)	.507	(.436–.579)	.646	(.553–.739)	.699	(.628–.770)	.630	(.544–.716)
PROMIS 6-item	.578	.660	.634	(.542–.727	.570	(.493–.646)	.530	(.457–.603)	.638	(.542–.734)	.712	(.642–.782)	.631	(.546–.715)
PROMIS 8-item	.574	.664	.623	(.530–.716	.575	(.499–.650)	.523	(.450–.596)	.646	(.550–.741)	.712	(.646–.785)	.633	(.550–.717)
PROMIS Short-form	.583	.671	.644	(.551–.736)	.577	(.501–.652)	.529	(.456–.602)	.638	(.543–.732)	.741	(.673–.808)	.634	(.551–.717)
PHQ-9	.617	.649	.697	(.608–.786)	.587	(.512–.662)	.566	(.493–.639)	.664	(.572–.755)	.644	(.570–.717)	.640	(.557–.723)
PHQ-2	.570	.627	.598	(.597–.690)	.576	(.505–.646)	.535	(.464–.606)	.576	(.483–.670)	.686	(.619–.752)	.620	(.537–.703)
SF-36 Mental Health	–	–	.627	(.534–.720)	–	–	–	–	.745	(.661–.829)				

*AUC is probability of correctly discriminating between patients who have improved and those who have not. Any improvement ≥ “a little better”; moderate improvement ≥ “moderately better”

^†^6 month follow-up for CAMEO; 3 months for SPACE and SSM. The proportion of patients reporting any improvement by *retrospective GRC *was 57%, 40%, and 57% in CAMEO, SPACE, and SSM, respectively. The proportion reporting any improvement by *prospective GRC* was 40%, 39%, and 29% in CAMEO, SPACE, and SSM, respectively

There were no significant differences at P < .01 (using Bonferroni’s correction for multiple comparisons) between any of the retrospective AUC’s. The prospective AUC was significantly lower for the PHQ-2 (P = .003) compared to the PROMIS Short-form in the CAMEO trial

### Agreement between retrospective and global rating of change anchors

The retrospective and prospective global change anchors agreed in their categorization of individuals as better, same, or worse in 68 of 136 participants in CAMEO, 123 of 223 in SPACE, and 95 of 238 in SSSM, resulting in simple agreement rates of 50%, 55%, and 40% respectively. The corresponding weighted kappas in the 3 trials were 0.228, 0.233, and − 0.027.

## Discussion

Using data from three clinical trials, we found PROMIS depression scales were responsive to change using both prospective and retrospective global change anchors as well as AUC analysis. Responsiveness was similar among all four fixed-length PROMIS scales and comparable to the responsiveness of the PHQ-9 and PHQ-2. In general, the measures were better able to detect depression improvement than worsening. A strength of our study compared to previous research on responsiveness of PROMIS depression measures is the triangulation of results from three patient samples using three measures of responsiveness.

Only a few prior studies have explored the responsiveness of PROMIS depression scales. In an observational study of 234 patients undergoing inpatient treatment in four psychosomatic rehabilitation centers, the pre-post treatment effect size was similar for the PROMIS depression item bank scale (using all 28 items) and the Center for Epidemiological Studies Depression scale (CES-D) (1.16 vs. 1.09) [7]. In a second observational study of 194 patients with depression treated for 12 weeks, the PROMIS CAT was similar to the PHQ-9 and CES-D in terms of treatment effect size: 0.84, 0.98, and 1.06, respectively [5]. However, depression recovery defined in several different ways was less frequent with the PHQ-9 compared to PROMIS and CES-D. In contrast, the PHQ-9 and PROMIS 8-item short-form had similar responsiveness in identifying depression recovery in a longitudinal study of 701 patients with neurological or psychiatric disorders [8]. In a longitudinal study of 903 patients with 5 diverse diseases (4 medical conditions and major depressive disorder), two thirds of patients completed PROMIS by CAT and one-third with an 8-item short form [9]. The average SRM using a retrospective global anchor was 0.71 for the improved group and − 0.49 for the group that worsened. In a longitudinal study of 150 patients with depression, SRMs in those experiencing recovery were 0.82 and 0.79 for the PROMIS 28-item bank and 8-item short form depression scales, respectively, and 1.00 for the PHQ-9 [6]. Unlike these previous studies that used either an observational design, a single sample, or PROMIS administration by CAT or the entire item bank, we used data from three RCTs and evaluated four PROMIS short forms of varying lengths. In addition, we evaluated responsiveness by triangulating several methods. Thus, our study substantially strengthens the evidence regarding the responsiveness of PROMIS depression scales.

Responsiveness was not symmetric with respect to improvement and worsening. SRMs for improvement averaged a moderate positive effect size and were roughly twice the SRMs for worsening which averaged a small negative effective size. Also, the 3 to 6 point improvement in PROMIS depression T-scores was above the minimally important difference. This greater sensitivity of symptom scales for detecting improvement has been previously reported for depression [5, 16, 24], pain [20, 22, 25–28] and anxiety [24].

The Consensus-based Standards for the selection of health Measurement Instruments (COSMIN) guidelines consider SRMs and other effect size metrics an imperfect approach to assessing responsiveness [29] and also discuss the limitations of transition anchors such as global rating of change. Objections to these opinions [30] as well as the COSMIN rationale [31] have been subsequently articulated. Suffice it to say, SRMs and effect sizes as well as global of rating change anchors have been widely used to assess responsiveness both before [3, 30, 32–34] and since [20, 35–42] publication of the COSMIN guidelines; only a small number of representative studies are cited here.

The AUCs in Table 4 represent modest rather than strong differentiation between patients whose depression had improved and those who were the same or worse. However, AUCs have been reported in a similar range in other studies using retrospective global rating of change as an anchor [16, 20, 27, 43] in which AUCs tend to be lower than in studies of diagnostic tests for which there is a criterion (“gold”) standard to determine the presence of a disease. Retrospective global ratings of change may be influenced by recall bias as well as the current state of symptoms [19, 44]. Some experts recommend an AUC ≥ 0.70 as a threshold for responsiveness when using a criterion standard anchor but also acknowledge that criterion standards often do not exist for patient-reported outcomes (PROs) [29, 45]. Thus, AUCs for scales measuring symptoms and other PROs have been < 0.70 not only when using retrospective global change anchors but also in some studies using other anchors as well [32, 46, 47]. Ours is the first study to also use prospective global change anchors to assess AUCs for PRO scales. Although this anchor lead to more AUC estimates ≥ 0.70, the sample size of those with moderate change by this anchor was small yielding wide confidence intervals. For all these reasons, the similarity of AUCs when using a global change anchors is more salient than their absolute value [48].

Scale length did not have a strong effect on responsiveness. The four PROMIS depression scales ranging from 4 to 8 items had similar responsiveness, a finding previously reported for PROMIS pain scales [20]. The PROMIS fixed-length scales for a specific domain share some items in common, which may explain in part their comparable responsiveness. Also, the average responsiveness of the PHQ-9 and PHQ-2 did not differ substantially, as has been shown in only one previous study [13]. Short measures may be more desirable for studies with many outcome measures, particularly where depression is a secondary rather than primary outcome, or in busy clinical practice settings with time constraints or the need to assess multiple patient-reported outcome measures.

Methodologically, our study is relatively unique in using both retrospective and prospective global change anchors allowing assessment of responsiveness with two different global anchors. Notably, two of the trials only showed fair agreement beyond chance of these two anchors in classifying individuals as better, same or worse, and one trial showed poor to no agreement beyond chance. It is possible that the two anchors provide different perspectives of change over time. Alternatively, it may be that one anchor is superior to another or that both anchors have limitations, but this would require additional research comparing both anchors to a third independent anchor. However, as already discussed, criterion standard anchors for patient-reported outcomes are lacking. Moreover, global rating of change is among one of the most commonly-used anchors for assessing responsiveness.

Our study has several limitations. First, depression was generally mild in all three samples, thereby restricting the range in which depression improvement could be detected. Responsiveness needs to be further studied in more clinically depressed samples in which treatment is warranted and a responsive measure is especially important. Second, because the samples included predominantly male veterans with either chronic pain or stroke, findings need to be replicated in populations with more women and a broader range of medical and mental health conditions. Third, one legacy measure (SF-36 Mental Health) was used only in one trial (CAMEO). Although its responsiveness has been demonstrated in prior studies, its comparative responsiveness to the PHQ-9 and PROMIS scales requires additional testing. Fourth, because we made multiple statistical comparisons between depression measures, the differences between measures should be interpreted cautiously unless highly significant (i.e., p < 0.001). Fifth, the nested nature of the PROMIS scales (i.e., sharing many items in common) as well as the PHQ-2 items being included in the PHQ-9 would lead to some convergence of responsiveness within the same family of scales. Sixth, studies using additional responsiveness metrics besides SRMs anchored to global ratings of change are warranted. Finally, our findings are derived from secondary analyses of data from clinical trials rather than a primary hypothesis-driven psychometric study.

## Conclusions

Two well-validated and widely-used depression measures—the PHQ-9 and PROMIS scales—have generally comparable responsiveness. Moreover, the shorter versions of these scales also appear responsive. Our findings provide initial evidence of responsiveness which should be further tested in other patient samples using additional responsiveness metrics. The fact that both measures are public domain and available in numerous translations are additional advantages. Because measures seem better in detecting improvement than worsening, calculating the change in score together with a single question about global change may be desirable to optimize recognition of deterioration in symptom-based conditions like depression and pain. Recent initiatives to incorporate depression and other patient-reported outcome measures into routine practice as well as embedding them in the electronic health record will further enhance symptom detection and management.[49].

## Data Availability

Datasets and analyses codes generated during the current study are available from the corresponding author on reasonable request.
